# Foreign Summary

**Published:** 1839-01-12

**Authors:** 


### FOREIGN SUMMARY.

*Death of Broussais.*—PROFESSOR BROUSSAIS died at one o'clock on Sunday morning, the 18th November, at his country seat, of Vitry, a few miles from Paris.

His immediate decease was rather sudden, but he had long laboured under cancer of the rectum.

Broussais was born at St. Malo, in December, 1772, and was therefore sixty-six years of age when he died. In 1792 he entered the army as a private soldier, but soon afterwards became an *officier de santé.* He subsequently served in a trading vessel during a period of six years, after which he went to Paris, and graduated as Doctor in Medicine. His thesis was on Hectic Fever, and was dedicated to Pinel.

Subsequently to this, he followed the campaigns in Holland, Germany, and Spain; and it is said to have been amid the fatigues of military service that he conceived the plan of the work to which he owes his celebrity—the History of Chronic Phlegmasiæ. Of this the fifth edition was published in Paris during the current year.

Broussais was Physician-in-Chief to the Val-de-Grace; Professor of General Pathology in the Ecole de Médecine; and a Commander of the Legion of Honour. His appointments brought him 10,000 fr. per annum.

He was attended in his last illness by M. Amussat, and when arrested by death, was actively engaged in a reply to the Memoir of M. Jouffroy against Phrenology, and in preparing a new edition of his work on Irritation and Insanity. There was a rumour, arising probably from the abruptness of his death, that he had been poisoned; but there seems to have been no ground for such a suspicion, and it appears to have speedily subsided.

M. Broussais was buried on the 21st of November, on which occasion all the usual display and parade which mark such scenes in Paris, were exhibited. A crowd of practitioners and pupils were assembled in the Rue d'Enfer; military medical officers, and the members of the Ecole, in their official dresses; deputations from the Academies of Sciences and of Medicine, were in attendance, to say nothing of a detachment of troops. This imposing *cortège* proceeded to the Val-de-Grace, MM. Larrey, Orfila, Boissy d'Anglas, and Droz, being the pall-bearers. Divine service having been performed in the chapel, the procession proceeded, the students having taken out the horses, and dragging the hearse all the way to Père-la-Chaise.

Discourses were pronounced over the grave by MM. Droz and Arago, in the name of the Institute; M Larrey (*fils*) on the part of the military medical officers; and M. Bouillaud on that of the Ecole de Médicine.

The officers at the Val-de-Grace propose to go into mourning for a month, as a testimony of their affectionate respect for the deceased.

A subscription has been opened in Paris, for the purpose of erecting a monument to the deceased.—*Lond. Med. Gaz. Dec.* 1, 1838.

*Cauterization of the Pharynx in Croup.*—It appears this remedy was first tried by Dr. Peronneau in the treatment of laryngeal and pharyngeal inflammations, and Dr. Hatin has since used it in four cases of incipient croup [?] with complete success. The first case given is that of a child five years of age, who was seized with that peculiar hoarse cough so difficult to compare, but so easily recognised when once heard, and which indicates the commencement of croup. Leeches were immediately applied; and on fears being expressed to the father regarding the termination, he related the case of a child who had been cured of an attack of croup by cauterization practised by M. Peronneau. M. P. was then called to the present case, and the following course was adopted. The child was placed upon the father's knees, who with one hand fixed the arms, and with the other retained the head against his chest. The operator placed himself in front, holding in his left hand an instrument necessary to keep the mouth open and to depress the tongue, and in the right a *porte-pierre*, bent like a sound, and containing a piece of nitrate of silver, projecting some lines. The tongue being depressed, the tube containing the nitrate of silver was passed into the posterior fauces and rapidly passed over all points for a second or two; the two instruments were then withdrawn to allow of respiration. Some minutes after, a second cauterization, similar to the first, finished the operation. The child did not complain of much pain or uneasiness. The next morning, after a quiet night, the cough was simply catarrhal, and the patient out of danger. Upon looking into the throat, the tonsils, soft palate, posterior wall of the pharynx, and all the points accessible to the sight were covered with a white eschar, which after a few days disappeared, leaving a bright red appearance, not causing sufficient pain to embarrass deglutition.

Another case is related occurring in the son of M. Imard, director of the Hospital "La Pitié." This child, nine years and a half old, was seized in the night with the premonitory symptoms of croup, and Dr. Hatin was summoned in the morning. He at once determined to practise cauterization, but first requested the advice of M. Serres, whose opinion was similar to his own. The application was made in exactly the same way as the last case. In the evening the croupy cough had disappeared, and that which remained gradually dimished in intensity.

Two other cases are given in which this method of treatment was equally efficacious. To ensure success, it is necessary that the cauterization should be performed during the first few hours of the attack, for if the false membrane occupy the larynx or trachea, the application will be ineffectual, at least Dr. H. has employed it in two such cases, and it exerted no influence on the progress of the disease.

*Brit. For. Med. Rev., from Revue Medicale.*

---

*Dislocation of the Humerus, attended with a grating Sensation on Motion, leading to the supposition that the case was complicated with Fracture.* By William Lawrence.—James Yarnsley, forty years of age, was admitted into the hospital, on the 23d of March, 1838, for an accident to the shoulder, which had occurred on the 21st. A cart, in which he was riding, was overturned; he was thrown violently to the ground, when the cart fell on him, and he remained under it for some time. The gentleman, who first examined the limb, considered that there was a fracture, and therefore recommended that he should be sent from the country, where the accident happened, to the hospital. They who first examined the patient on his arrival entertained the opinion that there was fracture; and the case was accordingly mentioned to me as a dislocation of the shoulder with fracture. The dislocation was obvious enough, and it was soon ascertained that the humerus was not broken. A sensation like crepitus was perceived as distinctly as in a fracture, when the shoulder-joint was firmly grasped with one hand, and the arm moved with the other; also, when the upper end of the bone was raised by the hand passed under it in the axilla, the elbow being held by the other hand. The sensation appeared to me more like the hitch or catch which might be produced by moving the articular head of the bone over an irregular hard surface, than the sharp grating of broken bones: the symptom, however, was so strongly marked, as to lead to the opinion that the neck of the scapula was fractured. Never having seen a specimen of fractured neck of the scapula in any museum, and reflecting on the mode in which this portion of the bone is protected against external violence, I conclude that such an injury, if it ever happen at all, is extremely rare, and that it is the least likely to take place when the effect of the force has been spent in causing dislocation. As the existence of dislocation was unequivocal, while I doubted altogether respecting that of fracture, I deemed it advisable to make a cautious trial of extension, which I did on the 24th. When a moderate force had been applied, by two or three assistants pulling at the ends of a folded linen fastened above the elbow not more than five minutes, the bone went in, the mobility of the joint was restored, and there was no longer any crepitus or other indication of fracture.

The head of the humerus, when dislocated, may lie upon the subscapularis, or between that muscle and the bone; or it may be placed in contact with the inferior costa of the scapula, near the glenoid cavity. In the two latter cases, the movement of the head over the bony surfaces, on which it rests, may impart a sensation closely resembling the crepitus of fracture. I remember a case of unreduced dislocation in this hospital, where the crepitus was so distinct that the injury was supposed to be fracture. The patient died: I do not recollect the details of the history, nor the cause of death. The head of the humerus was in contact with one of the ribs, the surface of which was bare.—*Lon. Med. Gaz.*

---

*Creosote in Deafness.* By J. Harrison Curtis.—One of the principal, and most common, causes of deafness is a deficiency of the secretion, from a want of action of the ceruminous glands. Many cases of loss of hearing which have been under my care, even when the disease has been of long standing, have been fairly referrible to this cause, and, on its removal, the deafness has disappeared; of course, in a longer or shorter time, according to the previous duration of the disease, and the severity of the primary cause of the inaction of the glands. When the meatus auditorius has been duly cleansed,* and the orifices of the ducts have been, as it were, re-opened, by the removal of the diseased secretion by which they where occluded, a moderate stimulant is of essential service in restoring the glands to healthy action; but the cleansing is impera-

* Physiologists are of opinion that there are not any muscular fibres in the meatus by which, under any circumstances, the cerumen is discharged; but it has been conjectured that its expulsion is effected by the motions of the lower jaw during mastication.

tively necessary, and no remedy will be of use until that operation has been properly performed. I generally employ a preparation consisting of half an ounce of ox-gall, mixed with a drachm of tincture of castor, or tincture of musk, with which a piece of cotton is moistened, and inserted into the meatus at night, to soften the hardened cerumen, the ear being syringed in the morning with warm water, to which one ounce of soap liniment and a little eau de Cologne, have been added. I occasionally substitute the solution of potash of the Pharmacopœia, with oil of almonds, for the preparation of ox-gall and castor, with equal advantage in dissolving the cerumen. In effecting this, it is necessary to be particular in the choice of a syringe. When this important object has been obtained, and the ducts and glands are in a fit state to be acted on by the stimulant, a solution of creosote in oil of almonds, I am led to believe, from experience, will be found to be of great advantage in inducing the ceruminous glands to resume their healthy action. The formula I use is as follows:—Take of

*Creosote*, one drachm;

*Oil of almonds*, four drachms. Mix; a little to be inserted into the meatus, night and morning, with a camel-hair brush.

I generally commence with a solution of this strength, occasionally gradually increasing the quantity of creosote employed, according to the effects produced. Cases, however, have occurred, as will be shown, in which this application has not been beneficial, until after the previous employment of blisters behind the ears, of an ointment made with tartarised antimony, or of other derivatives, which are required to abate any irritation to which the ear may be subjected. In cases of otorrhœa, or where there is any pain or inflammation, its use is contra-indicated. Its application does not cause any pain or smarting sensation, the only sensible effect produced being a feeling of agreeable warmth.—*Lancet.*

---

*Dr. Graves on the treatment of Epistaxis.*—Permit me now, gentlemen, to direct your attention to the treatment of one form of bleeding from the nose. It not unfrequently happens that epistaxis constitutes the only ailment to which young persons are liable. I was consulted by two gentlemen within the last year, the one eighteen, the other twenty-eight years of age; they were both healthy in every other respect, and were both liable to bleeding from the nose, sometimes slight, sometimes copious, and then producing a degree of debility proportionate to the extent of the hæmorrhage; no disturbance of the digestive organs, of the heart, or of any viscus or function, was discoverable. There seemed to be but one defect in the constitution, scarcely explicable except on the somewhat mechanical hypothesis of a superabundance of blood, accompanied, perhaps, by a defect in the process of sanguification, whereby the blood's fluidity was altered. These ideas, borrowed from the now antiquated humoral pathology, served to indicate the method of treatment; and having no better guide to follow, I proceeded to put the plan thus suggested into execution: I accordingly advised my patients to live as dry as possible, or in other words, to restrict themselves to a minimum of drink. I directed them at the same time to take about half a drachm of dilute nitric acid daily, in divided doses. Although the reasoning which led to its adoption is scarcely tenable, yet the remarkable success of the treatment renders the result worth recording.

Hippocrates, in his curious and instructive work on diet, insists much on attention being paid to the quantity of drink allowed to patients in different diseases; it is singular, however, that he nowhere speaks of restricting the quantity of drink in cases of hæmorrhage.

Dr. Williams has lately recommended the dry treatment in catarrhal affections of the lungs attended with increased secretion. In young persons, when the sputa are abundant and easily gotten up, I can attest the efficacy of an almost total abstinence from drink. Not long ago, I was called to see a young lady, then on a visit in the house of the venerable Doctor Percival: she had been blistered, and had taken large quantities of squills, ipecacuanha, antimonial wine, and other expectorants, and had refrained from solid food, and indulged freely in demulcent ptisans, whey, tea, &c.; these means, with confinement to her room, had been continued about a week without the slightest benefit; the cough was incessant, depriving her altogether of sleep, and accompanied with much wheezing, and an abundant easy expectoration. All remedies were laid aside, an almost total abstinence from drink observed, aud a strikingly rapid cure effected. In his work on Diet, Hippocrates gives some hints worth attending to; thus, in cases of constipation, he recommends a very varied diet, and he does so on good grounds, for a simple uniform diet is very apt to occasion constipation. Hippocrates lays much stress on different sorts of exercise in different states of health: riding, walking, running, wrestling, and rolling in the dust, &c. &c., are all examined, at large, and recommended as suitable to particular conditions of the constitution; but running, riding, and walking, have also their specified varieties, not merely as to duration and velocity, but as to direction, for he carefully distinguishes locomotion according as it is continued in straight lines, in curves, or in greater or less circles, on flat, or on hilly ground, &c. &c. Exercise, in curves or in circles, appears to have been a favourite gymnastic remedy among the Greeks; it is now quite neglected, but perhaps undeservedly, for running, riding, or walking, in curves or circles, must bring a number of muscles into play, which are comparatively unemployed in rectilinear progression. The effects on the circulating and nervous systems must be likewise different, as is evident from the remarkable disturbance they undergo in the circular swing.—*Lond. Med. Gaz.*

---

*Adulteration of Quinine.*—M. Pelletier, of Paris, states that if twenty drops of the pure and concentrated sulphuric acid be poured upon twenty grains of suspected quinine, the solution will present a most beautiful crimson colour, more or less intense, according to the quantity of salicine present. The adulteration of one part of salicine with ninety-nine of quinine is, by these means, easily discovered.—*Lancet.*

---

*Amputation of the Neck of the Uterus.*—M. Retzius, of Stockholm, performed this operation, during the year 1832, three times,—but, on each occasion, the carcinomatous affection returned, and extended to the rest of the uterus. His experience is opposed to that of M. Lisfranc, and, from the numerous examinations of the dead body which he has made, he is convinced that carcinoma is rarely confined to the neck of the uterus.—*Ib.*

[M. Lisfranc's statements upon this subject have been contradicted by the interne of his hospital, whose assertions render it quite probable that there is no actual opposition in the experience of the Parisian and Swedish surgeons.—Eds.]

---

*Extensive Desquamation.*—A patient for some time subject to attacks of fever, had, besides the common febrile symptoms upon the invasion of the disease, universal itching of the skin, and more especially at the joints; and the itching was succeeded by a number of little red spots, with a slight degree of swelling. Soon after this his fingers became very stiff, hard, and painful at their ends, and at the roots of his nails. In twenty-four hours, or thereabouts, the cuticle began to separate from the cutis, and in ten or twelve days this separation was general from head to foot, when he has many times turned the cuticle off from the wrists to the fingers' ends completely, like gloves; and in the same manner also to the ends of the toes; after which his nails shoot gradually from their roots, at first attended with exquisite pain, which abates as the separation of the cuticle advances, and the nails are generally thrown off by new ones in about six months. The cuticle rose in the palms of his hands and the soles of his feet like blisters, but contained no fluid under them; and when it came off, left the subjacent skin very sensible for a few days.

Sometimes upon catching cold before he has been quite free from feverish symptoms, he has had a second separation of the cuticle, but then so thin as to appear only like scurf; thus demonstrating the quick renewal of this part.—*Lon. Med. Gaz.*

---

*Dr. Graves on Chilblains.*—Many persons, especially children, suffer much from chilblains, although this troublesome affection is often met with in the most healthy constitutions; yet, when the disease proceeds to a very great extent and degree of intensity, and occurs with violence, where the exciting cause, exposure to changes of temperature, has not been sudden or remarkable, we may then conclude that the sufferer's diathesis is decidedly scrofulous. This affection ought consequently to excite the attention of parents; for although in general it is merely a local ailment, yet in some children it indicates a general weakness of the constitution, and in all occasions much pain and annoyance. Sir Benjamin Brodie, by his admirable observations on the nature and cure of corns, published in the 17th volume of the Medical Gazette, has shown that affections, vulgarly reputed to be beneath the dignity of the medical profession, may afford a legitimate and ample field for our interference and assistance. In order to prevent the formation of chilblains, we must endeavour to protect the skin from the operation of the usual exciting cause of the disease, and, in addition to cautioning the children to avoid exposing their hands or feet to rapid transitions from cold to heat, we should endeavour to render the skin capable of bearing moderate changes of temperature with impunity. This is best effected by washing the hands several times a day, at first with tepid and afterwards with cold water, mixed with a small proportion of spirits or of *eau de Cologne.* Some parents do much injury by making their children wear flannel or woollen gloves, even in the house. Stimulating liquids, such as strong brine, have long been deservedly popular as preventives of chilblains, and were recommended by Dioscorides; but none of those usually employed seem to me as efficacious as one which I was the first to use, viz. a solution of sulphate of copper in water, in the proportion of ten grains to the ounce. This must be diligently applied to affected or suspected parts of the skin with a camel's hair pencil; and as soon as the moisture dries off, the skin should be well smeared over with spermaceti ointment. The sulphate of copper lotion may be applied two or three evenings in succession, until it has produced a manifest effect on the skin; it must be then discontinued for a few nights—again, however, to be resumed as soon as the natural soft and tender texture of the skin seems about to return. You must be careful to enjoin the application of the spermaceti after each use of the lotion By this simple plan, commenced early in winter, many children, previously martyrs to chilblains, have been completely protected. It is probable that the nitrate of silver would answer equally well, did it not discolour the skin in so unseemly a way.—*Ib.*

---

*Subcarbonate of Iron in Hooping-cough.*—In No. 18, vol. 1st, we published an article on the use of the subcarbonate of iron in hooping-cough, by Dr. Steymann. In the November number of the Dublin Journal of Medical Science, Dr. Lombard, of Geneva, confirms the statements of Dr. Steymann, and announces that he found the remedy a specific, in a late epidemic of hooping-cough at Geneva. He pushed the medicine to doses of twenty-four, and even thirty-six grains a day, in young children, and the result of his experience was, "that it enjoys a remarkable property to make the fits less violent, to diminish their number, and, after a certain number of days, to cure entirely the hooping-cough."

